# Insulin-induced Lipohypertrophy in Patients with Type 1 Diabetes Mellitus Treated with an Insulin Pump

**DOI:** 10.1155/2022/9169296

**Published:** 2022-01-24

**Authors:** Damian Ucieklak, Sandra Mrozinska, Aleksandra Wojnarska, Maciej T. Malecki, Tomasz Klupa, Bartłomiej Matejko

**Affiliations:** ^1^Department of Metabolic Diseases, Jagiellonian University Medical College, Krakow, Poland; ^2^University Hospital, Krakow, Poland; ^3^Department of Radiology, Jagiellonian University Medical College, Krakow, Poland

## Abstract

**Background:**

Lipohypertrophy (LH) of subcutaneous tissue is an insulin-induced complication occurring in patients with diabetes. We aimed to define the prevalence of LH and identify its risk factors in type 1 diabetes (T1DM) patients treated with continuous subcutaneous insulin infusion (CSII).

**Materials and Methods:**

The study included 79 consecutive CSII-treated T1DM patients. The diagnose of LH was based on ultrasonography (US) as a reference method, physical examination was also performed. Clinical characteristics were available from the medical records.

**Results:**

The median age of patients was 28 years (interquartile range [IQR], 24–30.5) with a body mass index (BMI) of 24.5 ± 3.5 kg/m^2^, HbA_1c_ 7.1% (IQR, 6.7–8.1), T1DM duration 15 (9–20) years, and CSII use duration of 8 year (IQR, 5–11). LH was detected by US in 75 (94.9%) patients. This value was much higher than this obtained by visual assessment (*n* = 39, 49.4%) or palpation (*n* = 59, 74.7%). In univariate analyses, the following risk factors for occurrence of 5 and more LH lesions were identified: the ratio of insulin dose to body mass exceeding 0.7 IU/kg (OR, 3.69; 95% CI, 1.43–10.01) and the total daily insulin dose (OR, 1.05; 95% CI, 1.02–1.09). A higher dose of insulin per kg remained a significant risk factor of LH amount in multivariate analysis.

**Conclusion:**

This selected T1DM cohort treated with CSII had a very high prevalence of LH. US assessment should be considered as a reference method for LH screening in T1DM patients. The identified risk factors for the number of LH lesions were related to insulin dosing.

## 1. Introduction

Lipohypertrophy (LH) is a common complication of insulin therapy in subcutaneous tissue observed in patients with diabetes [1]. It manifests as soft benign nodules on the skin surface [1]. LH occurrence is associated with the lipogenic action of insulin at the site of injection and repeated trauma related to performing insulin injections at the same site [2]. Several risk factors of LH such as a needle reuse, a lack of rotation of injection sites, a small size of rotation area, a high daily insulin dose, a long duration of insulin treatment, a high body mass index (BMI), poor glycemic control, and low level of patient education [3–6] were reported from clinically diverse diabetic cohorts. The risk of LH in patients treated with multiple daily insulin injections was reported to be lower when using insulin analogs than human insulin [2]. The insulin absorption from affected areas is impaired and can be probably attributed to the local inflammatory reaction, increased local fibrosis, and decreased vascularity [7–9].

Of note, LH has been linked to poor glycemic control as well as a higher frequency of unexplained episodes of hypoglycemia as well as an increased variability in glucose levels and ketoacidosis [10–13]. What is more, injecting insulin into LH lesions can lead to an inappropriate increase in insulin dosing and-consequently higher costs of insulin therapy [14,15]. Omitting during injections places affected by LH seems to decrease total insulin dose and improve glycemic control [16–18].

Data on the prevalence of LH varies significantly, depending on the characteristics of the study group, method of insulin therapy, and methodology used for LH diagnosis [14, 19–21]. Ultrasonography (US) was recognized to be a useful diagnostic method for the assessment of LH [7, 20, 22]. The typical ultrasound echo representation of LH is an increased echogenicity spot in diffuse areas of the subcutaneous tissue where insulin is injected. However, LH can sometimes manifest as clearly defined nodules with circumscribed margins [20]. Hyperechogenic areas have a prevailing fibrotic component while hypoechogenic ones have edema or fluid components without fibrotic segments [7]. Another important feature of LH nodules that helps differentiate LH lesions from other benign superficial masses like lipomas, hematomas, or fluid-filled cysts [22] is the lack of a capsule or vascularity. In this study, we aimed to define the prevalence of LH in T1DM patients treated with continuous subcutaneous insulin infusion (CSII) on basis of US as a reference method. A second goal was to identify risk factors.

## 2. Materials and Methods

We evaluated 79 consecutive T1DM patients treated with an insulin pump from the outpatients of Department of Metabolic Disease, University Hospital in Krakow (tertiary diabetes reference center). The following inclusion criteria must have been fulfilled: (1) diagnosis of T1DM according to WHO criteria [23]; (2) age at the examination 18–50 years; (3) insulin pump use for at least 6 months; and (4) having given written informed consent. Exclusion criteria were as follows: (1) pregnancy, (2) BMI >32 kg/m^2^; (3) atopic dermatitis; (4) subcutaneous administration of medicines other than insulin in the last 6 months; and (5) presence of advanced diabetes complications such as end-stage renal disease or loss of vision.

Clinical characteristics of the patients were obtained from their medical records. The data from the insulin pump reports and glucometers for the last 14 days before the examination was collected. Patients were asked to complete a questionnaire about the frequency of changing the infusion sets.

All patients were examined for the presence of LH by the physical examination through the visual assessment and palpation and with US (Acusone, Siemens) scanning. US examination of all patients was performed using one of the two sonographers participating in this study. On US, LH was diagnosed based on previously described criteria [22]. These involved lesions characterized by: (a) localization in the subcutaneous tissue between the *epidermis* and the muscle layer in the area of the insulin injections; (b) clear circumscription either by hyperechoic foci with defined borders or a nodular shape with a hypoechoic halo; (c) heterogeneous echotexture versus surrounding tissue; (d) distortion of surrounding connective tissue; and (e) absence of a capsule or vascularity.

The number and localization of LH lesions that were found in each examination and the largest dimension of the biggest LH lesion on US were recorded. Considering the limitations of LH severity assessment based only on the number of lesions, we also proposed a new LH severity scale considering both the amount and size of LH lesions. The details of scoring were as follows:

Number of LH lesion: none = 0 points; 1 lesion = 1 point; 2–3 lesions = 2 points; 4–7 lesions = 3 points; and ≥8 lesions = 4 points.

Size: no LH lesion = 0 points; the maximal diameter of the biggest LH lesion ≤1 cm = 1 point; > 1 and ≤ 2 cm = 2 points; > 2 and ≤ 3 cm = 3 points; and >3 cm = 4 points.

The sum of the number and size points together categorized LH into four stages of advancement: no LH = 0 points; mild LH = 1–2 points; moderate LH = 3–5 points; advanced LH = 6–8 points.

Visual inspection and palpation of insertion sites were performed in patients in both a standing and supine position. In the visual assessment, lesions that protruded above the skin surface were rated as LH. Other skin lesions like spot marks (i.e., slight redness, scars) in areas of the former cannula implantation in the level of skin surface were not counted as LH. The definition of LH in palpatory examination was based on earlier publications [22]. Briefly, LH was defined as discrete palpable dermal nodules or swellings of variable consistency. These were either thickened, had a ‘rubbery' texture, or felt firm and were located within the subcutaneous tissue at insulin injection sites. The term “subclinical LH” was reserved for LH lesions detected on US which were not detected by visual or palpation method [22]. All participants provided written informed consent in accordance with the Declaration of Helsinki.

## 3. Statistical Analysis

Continuous variables with normal distribution were expressed as the mean ± standard deviation (SD). Continuous variables not normally distributed were presented as median and interquartile range (IQR). The Shapiro-Wilk test was performed to assess whether a variable was normally distributed. The differences between two groups were assessed using the Student's or the Welch's *t*-test for normally distributed continuous variables; otherwise the Mann–Whitney *U* test was conducted. The Chi-square or Fisher's exact test was used to determine whether there is a significant association between two categorical variables. Three groups were compared using the Kruskal-Wallis test as non-parametric alternative to the one-way ANOVA test. The Pairwise Wilcoxon Rank Sum Test with Bonferoni correction was performed to calculate pairwise comparisons between group levels with corrections for multiple testing.

Uni- and multivariate logistic regression models were used to identify the risk factors of the number and the severity of LH lesions based on the proposed scale. In univariate models, we used the following variables: age, gender, BMI, T1DM duration, time on CSII, changing infusion sites regularly (Yes/No), changing infusion set every three days or more frequently (Yes/No), length of cannula above or equal to 9 mm, HbA_1c_ level, daily dose of insulin, and daily insulin dose above 0.7 IU/kg. A daily insulin dose above 0.7 IU/kg was chosen because this value constituted a mean insulin requirement in a large group of adult T1DM patients treated with CSII [24]. Variables with *p*-values less than 0.10 in the univariate model were used in multivariate analysis.

The Cohen statistic was used to assess the inter-observer agreement between LH lesions detected on US and physical examination. The McNemar test was used to check if two methods are equal in LH detection. We also assessed sensitivity and specificity of physical examination against US as a gold standard approach. The level of significance for the two-sided tests was set to below 0.05. Statistical analyses were performed using Statistica 12.5 software (StatSoft Inc., Tulsa, Oklahoma, United States) and R ver. 3.6.3 [25].

## 4. Results

### 4.1. Patient Characteristics

We included 79 consecutive T1DM patients [39, (49.4%) women]. Detailed clinical characteristics for the entire group as well as for subgroups with in-target of HbA_1c_ <7% and above-target HbA_1c_ levels are shown in Table 1. This includes a median age of 28 (IQR 24–30.5) years, a HbA_1c_ level of 54 (IQR 51–65) mmol/mol, 7.1% (IQR 6.7–8.1%), BMI 24.5 (±3.5) kg/m^2^, T1DM duration 15 years (IQR 9–20), and duration of insulin pump use of 8 years (IQR 5–11). Only six patients presented chronic diabetic complications (three cases of retinopathy, three of polyneuropathy, and one of diabetic kidney disease). At this sample size, the proportion of patients with ≥5 LH lesions on US was not statistically different between the two T1DM subgroups defined on the basis of glycemic control. However, this percentage was numerically higher in patients with the HbA1c level above the glycemic target (58.3 vs. 48.3%).

Additionally, we also collected data referring to the type of insulin, insertion cannula, pump model and pump manufacturer used by the patients. All patients were treated with rapid-acting insulin analogs: 52 (65.8%) patients with insulin lispro, 21 (26.6%) patients with insulin aspart, and six (7.6%) patients with insulin glulisine. The patients were using the following models of insulin pump: MiniMed Paradigm 715 (Medtronic)—*n* = 16 (20.2%), MiniMed Paradigm 722 (Medtronic)—*n* = 28 (35.4%), Medtronic MiniMed Veo (Medtronic)—*n* = 9 (11.4%), MiniMed 640G (Medtronic)—*n* = 7 (8.9%), Accu-Chek Spirit Combo (Roche Diabetes Care)—*n* = 18 (22.8%), and Dana (SOOIL)—*n* = 1 (1,2%). Most of the patients were using a Teflon cannula (*n* = 71, 89.9%), only a small subgroup employed a steel cannula (*n* = 7, 8.9%) (including three patients who used a Teflon and steel cannula interchangeably). The information about one patient was missing.

### 4.2. FH Assessment

LH was detected in 75 (94.9%) patients diagnosed with US, in 59 (74.7%) individuals LH was described by palpation and in 39 (49.4%) patients by visual assessment, and by physical assessment defined as visual and palpation combined in 63 (79.7%) patients (Supplemental Table 1). Two patients had none LH lesions found in all methods mentioned above. Also, 19 (24.1%) patients had skin lesions described as spot marks (redness, scars) in areas of former cannula implantation. We compared the subgroups of patients defined according to the number of LH lesions (<5 LH lesions and 5 or >LH lesions on US) as shown in Supplemental Table 2. Apart from a borderline result for BMI (23.7 ± 2.9 kg/m^2^ vs. 25.3 ± 3.9, respectively, *p*=0.05), there were no differences between the subgroups.

Subclinical LH was diagnosed in 61 patients (77.2%), who had some LH lesions detected on US but not on physical examination. In addition, 21 patients (26.6%) had some lesions reported as LH during the visual assessment and/or palpation that were not recognized as LH on US.

The most common area of LH localization was the abdomen wall: LH was found in 59 patients (74.7%) on US, in 45 (57%) by palpation, and in 34 (43%) by visual assessment. Two (2.5%) patients had subcutaneous atrophy in insulin-injected areas.

Overall, by visual assessment we detected 72 singular LH lesions, 134 LH lesions by palpation, and 372 based on US (*p* < =0.001) (Supplemental Table 3). There were 249 LH lesions detected only in US and not by visual or palpatory assessment. There were 29 LH lesions described in visual and/or palpatory assessment that were not visualized on US.

We identified 13 (16.5%) patients with the biggest dimension of LH below or equal to 1 cm, 30 (38.0%) patients had lesions greater than 1 cm but less or equal to 2 cm, in 10 (12.7%) patients LH size was more than 2 cm but less or equal to 3 cm, and in 22 (27.8%) patients the lesions were greater than 3 cm. On US examination, the majority of LH lesions featured hyperechogenic nodules-some with small hypoechogenic areas of edema or fluid. We found the ultrasound features of lipomas in five lesions described initially as LH in physical assessments.

US identified LH lesions more frequently than physical examination, respectively: 372 LH lesions vs. 152 LH lesions (*p* < 0.001). There was no inter-observer agreement between the number of LH lesions diagnosed during the physical examination and on US based on Cohen Kappa = −0.157 [95% CI, −0.24– −0.08]. Similarly, there was no inter-observer agreement between the number of patients diagnosed with LH in the physical examination and on US based on Cohen's Kappa = 0.129 [95% CI, −0.25–0.51]. The sensitivity of physical examination was 35.53% [95% CI, 30.51–40.8] (the US as a reference method), and its specificity was 0% [95% CI, 0.00–11.94].

We did not find any differences in the use of different insulin sets and rapid acting insulins between the groups defined on the basis of number and severity score of LH (Supplemental Tables 4 and 5).

### 4.3. Risk Factors of LH

#### 4.3.1. Risk Factors of LH Number

Univariate logistic regression models showed the following risk factors of developing five and more LH lesions visible on US: daily insulin dose above 0.7 IU/kg (OR, 3.69; 95% CI, 1.43–10.01), higher total daily insulin dose (OR, 1.05; 95% CI, 1.02–1.09), and higher BMI (OR, 1.14; 95% CI, 1.00–1.31–borderline significance). Multivariate logistic regression model revealed that a dose of insulin above 0.7 IU per kg remained a significant risk factor (OR, 3.37; 95% CI, 1.27–9.31) (Table 2).

#### 4.3.2. Risk Factors of LH Severity

The advancement of LH according to the proposed scale was as follows: no LH in 4 (5.05%) patients, mild LH in 4 (5.05%) patients, moderate LH in 41 (51.9%) patients, and advanced LH in 30 (38%) patients.

A univariate logistic regression model showed the following risk factors of advanced LH diagnosed by US: longer length of cannula (OR, 4.01; 95% CI, 1.52–11.39), daily insulin dose above 0.7 IU/kg of body weight (OR, 3.89, 95% CI, 1.47–10.96), higher daily insulin dose (OR, 1.03, 95% CI, 1.004–1.06), and higher BMI (OR, 1.22; 95% CI, 1.06–1.43). Multivariate logistic regression model revealed that the daily insulin dose above 0.7 IU/kg (OR, 4.81; 95% CI, 1.53–17.22) and length of cannula (OR, 3.44; 95% CI 1.11–11.74) were independent risk factors of the LH advancement (Table 3).

## 5. Discussion

This is the first study to assessing the LH of subcutaneous tissue based on physical examination and US in a selected cohort of young T1DM patients treated with a personal insulin pump. Scientific data on the LH in insulin pump users with T1DM is limited [26–29]. We confirmed previous findings from clinically diverse diabetic cohorts - LH is a common complication of insulin therapy in T1DM patients. We confirmed that for LH detection US is much more sensitive method than physical examination. Moreover, we defined several risk factors of LH number. Finally, we proposed a new scale for LH severity and assessed risk factors for advanced LH.

Previous meta-analysis reported that the prevalence of LH in patients with T1DM was 34% based on studies assessing LH by sight and palpation. In individual studies, this prevalence differed depending on patients' characteristic and the LH detection method [19, 20]. The LH prevalence assessed by palpation in studies involving patients on insulin pump therapy was of 44% [26]. Another study showed that 76% patients had LH assessed on US [29]. Our US-based research shows a markedly higher LH prevalence in T1DM patients treated with CSII and emphasizes the importance of LH screening in these patients.

The superiority of US over palpation was also associated with the fact that we could distinguish the LH from other subcutaneous lesions, e.g. lipoma. Some subjects had LH found with only US. These cases were undetectable in physical examination. Thus, the number of LH lesions in physical examination was substantially underestimated versus US as in previous studies [22, 30].

US seems to be currently a gold standard in LH detection [31]. One of the advantages of US examination over physical examination is the fact, that it enables precise assessment of LH size. Nevertheless, a physical examination is less time-consuming and less expensive than US and can be performed at every outpatient visit. Thus, the clinical value of these methods should not be underestimated.

We found that the dose of insulin per kilogram body weight was a LH risk factor on US - this agrees with other studies in which LH was associated with higher doses of insulin [2, 10, 32]. We also report for the first time that the insulin dose per kilogram was associated with LH severity.

The causative relationships in this observational study could not be established, but we have two potential explanations. First, insulin absorption in the area of LH is impaired, which forces patient to escalate insulin doses [9]. Alternatively, higher doses of insulin in the subcutaneous tissue caused LH. The length of cannula was another risk factor for LH severity. In previous studies involving patients treated with multiple daily injections, the needle length was reported to be a risk factor of LH in univariate analysis, but it lost significance in multivariate analysis [2, 3, 33]. There are no previous studies involving patients on insulin pump therapy in terms of association of cannula length and LH. No difference was found in insulin set or rapid acting analog use between the subgroups defined based on the number and severity score of LH. Of note, this was an observational study including one-point data and the number of patients in analyzed subgroups was rather limited. Thus, we are not able to definitely exclude the impact of specific insulin, equipment or supplies on the incidence of LH.

The BMI turned out not to be an independent risk factor of LH number and LH severity in multivariate models, and previous results are inconclusive [3, 7, 15, 32].

Some study limitations should be acknowledged. The sample size of this study is rather limited. Moreover, the causative relationships between variables could not be established due to the observational nature of this research. Additionally, T1DM duration differs substantially between the study participants; some patients are quite new to the pump. We also did not validate the clinical utility of the newly proposed severity scale. Finally, in spite of conducted LH severity assessment, a potential weakness of our study may be a lack of precise LH volume calculation in every patient.

The strength of our study is the homogeneity in the subjects in terms of the diabetes type, i.e., only CSII-treated T1DM patients were included. We also controlled for insulin analog use, severe chronic complications of diabetes, and obesity.

We suggest that assessment of LH on US should be first reserved to patients without LH lesions in clinical examination but with a high dose of insulin per kg. Moreover, patients should be educated about LH, its risk factors, consequences, and correct habits for infusion set insertion (site rotation, regular changing infusion sets, or omission of LH areas). Less than half of diabetes patients had declared being informed about the strategies how to prevent LH development [34].

In summary, this selected T1DM cohort treated with CSII had a very high prevalence of LH. US assessment can be a reference method for LH screening in T1DM patients. The independent risk factors of developing 5 and more LH lesions were related to insulin dosing.

## Figures and Tables

**Table 1:** Patients characteristics.

Variable	All patients (*n* = 79)^*∗*^	Patients with HbA_1c_ <7% (*n* = 30)	Patients with HbA_1c_ ≥ 7% (*n* = 48)	*P* ^*∗*^
Age, years	28 (24–30.5)	29 (24.3–33.8)	25.5 (24–30)	0.21
Men, *n* (%)	40 (50.6)	16 (53.3)	23 (47.9)	0.82
Diabetes duration, years	15.0 (9–20)	12 (9–19)	16.5 (8.8–20)	0.44
Time in CSII, years	8.5 ± 4.8	9.2 ± 5.1	8.2 ± 4.6	0.37
HbA_1c_, %; mmol/mol	7.1 (6.7–8.1) 54 (51–65)	6.5 (6.3–6.8) 47.5 (45.4–50.8)	7.7 (7.3–8.8) 60.7 (56.3–72.7)	<0.001
BMI, kg/m^2^	24.5 ± 3.5	24.2 ± 3.2	24.7 ± 3.7	0.54
MDI before insulin pump, years	3 (2–8)	2 (1–5.8)	4 (2–12.3)	0.01
Hypothyroidism, *n* (%)	14 (17.7)	5 (16.7)	8 (16.3)	0.99
≥5 LH lesions on US, *n* (%)	41 (51.8)	13 (43.3)	24 (58.3)	0.29

HbA_1c,_ hemoglobin A_1c_; CSII, continuous subcutaneous insulin infusion; BMI, body mass index; MDI, multiply insulin injection; LH, lipohyperthropy. ^*∗*^ For the comparison of patients with HbA_1c_ < 7% and ≥7%.

**Table 2:** Univariate and multivariate models for risk factors of 5 and more LH lesions.

Variable	OR per	Univariate		Multivariate	
		OR (95% CI)	*P*	OR (95% CI)	*P*
Age	1 year	1.003 (0.95–1.06)	0.91		
Male sex	No/Yes	0.95 (0.39–2.31)	0.91		
BMI	kg/m^2^	1.14 (1.00–1.31)	0.055	1.14 (0.98–1.33)	0.09
Diabetes duration	1 year	1.00 (0.94–1.06)	0.95		
Duration of CSII	1 year	1.02 (0.93–1.13)	0.60		
Regular changing of infusion sites	No/Yes	0.73 (0.21–2.44)	0.61		
Changing infusion set every 3 day or more frequently	No/Yes	1.35 (0.55–3.32)	0.51		
Length of cannula ≥9 mm	No/Yes	1.59 (0.65–3.96)	0.31		
HbA_1c_	%	1.23 (0.85–1.84)	0.29		
Dose of insulin >0.7 IU/kg	No/Yes	3.69 (1.43–10.01)	0.008	3.37 (1.27–9.31)	0.016
Dose of insulin	IU	1.05 (1.02–1.09)	0.002		

In the multivariate logistic regression model the variables with *p*-value <0.10 based on univariate logistic models were included (except for daily insulin dose because of including dose of insulin per kg body weight). OR, odds ratio; CI, confidence interval; for other abbreviations see Table 1.

**Table 3:** Univariate and multivariate models for risk factors of advanced LH based on the authorial scale (6–8 points).

Variable	OR per	Univariate		Multivariate	
		OR (95% CI)	*P*	OR (95% CI)	*P*
Age	1 year	1.04 (0.98–1.09)	0.18		
Male sex	No/Yes	1.29 (0.52–3.24)	0.58		
BMI	kg/m^2^	1.22 (1.06–1.43)	0.007	1.14 (0.96–1.37)	0.15
Diabetes duration	1 year	1.05 (0.99–1.12)	0.08	1.06 (0.99–1.14)	0.13
Duration of CSII	1 year	0.99 (0.90–1.09)	0.82		
Regular changing of infusion sites	No/Yes	0.67 (0.17–2.28)	0.53		
Changing infusion set every 3 day or more frequently	No/Yes	0.84 (0.33–2.11)	0.72		
Length of cannula ≥9 mm	No/Yes	4.01 (1.52–11.39)	0.007	3.44 (1.11–11.74)	0.038
HbA_1c_	%	1.11 (0.76–1.61)	0.57		
Dose of insulin >0.7 IU/kg	No/Yes	3.89 (1.47–10.96)	0.008	4.81 (1.53–17.22)	0.010
Dose of insulin	IU	1.03 (1.004–1.06)	0.03		

In the multivariate logistic regression model the variables with *p*-value <0.10 based on univariate logistic models were included (except for daily insulin dose because of including dose of insulin per kg body weight). For abbreviations see Tables 1 and 2.

## Data Availability

The data are available upon reasonable request from the corresponding author (b.matejko@uj.edu.pl).

## Supplementary Materials

Supplementary Materials Supplemental Table 1. The number of T1DM patients diagnosed with any LH lesion according to the diagnostic method. Supplemental Table 2. The comparison of the patients with less than 5 LH lesions and patients with 5 or more LH lesions on US. Supplemental Table 3. The comparison of the number of any lipohypertrophic (LH) lesions according to the diagnostic method in type 1 diabetes (T1DM) patients (*n* = 79). Supplemental Table 4. The comparison of the number of LH lesions and LH severity score according to the type of infusion set used by T1DM patients. Supplemental Table 5. The comparison of the number of LH lesions and LH severity score according to the insulin used by T1DM patients.
