# Supplementary material for: Insulin-induced Lipohypertrophy in Patients with Type 1 Diabetes Mellitus Treated with an Insulin Pump
Source: Int J Endocrinol. 2022 Jan 24;2022:9169296. doi: 10.1155/2022/9169296 (PMC8803446; doi:10.1155/2022/9169296)
Supplement: Supplementary Materials — Supplemental Table 1. The number of T1DM patients diagnosed with any LH lesion according to the diagnostic method. Supplemental Table 2. The comparison of the patients with less than 5 LH lesions and patients with 5 or more LH lesions on US. Supplemental Table 3. The comparison of the number of any lipohypertrophic (LH) lesions according to the diagnostic method in type 1 diabetes (T1DM) patients (n = 79). Supplemental Table 4. The comparison of the number of LH lesions and LH severity score according to the type of infusion set used by T1DM patients. Supplemental Table 5. The comparison of the number of LH lesions and LH severity score according to the insulin used by T1DM patients. [file 9169296.f1.pdf]

Supplemental Table 1. The number of T1DM patients diagnosed with any LH lesion according to the diagnostic method.

Region	Visual assessment	Palpation	Physical examination (visual assessment and palpation)	Ultrasonography	*P* *
All, n (%)	39 (49.4)	59 (74.7)	63 (79.7)	75 (94.9)	0.008
Abdomen, n (%)	34 (43)	45 (57)	51 (64.6)	59 (74.7)	0.23
Arms, n (%)	3 (3.8)	5 (6.3)	5 (6.3)	12 (15.2)	0.14
Buttocks, n (%)	2 (2.5)	10 (12.7)	10 (12.7)	20 (25.3)	0.07
Tights, n (%)	2 (2.5)	9 (11.4)	10 (12.7)	15 (19)	0.38
Subscapula, n (%)	-	-	-	1 (1.3)	0.99

*For the comparison of physical examination and ultrasonography.

Supplemental Table 2. The comparison of the patients with less than 5 LH lesions and patients with 5 or more LH lesions on US.

Variable	Patients less than 5 LH lesions or without LH on US, (n = 38)	Patients with 5 or more LH lesions on US, (n = 41)	*P**
Age, years	29.3 ± 8.4	29.5 ± 9.8	0.6
Men, n (%)	19 (50.0)	21 (51.2)	0.99
Diabetes duration, years	16.0 (8-20)	13.0 (10.0-20.0)	0.99
Time in CSII, years	8.2 ± 5.4	8.7 ± 4.3	0.41
HbA_{1c}, %; mmol/mol	7.0 (6.5-7.7)	7.2 (6.9-8.7)	0.25
	42.1 (47.5-60.7)	55.2 (51.9-71.6)	
BMI, kg/m^2	23.7 ± 2.9	25.3 ± 3.9	0.05
MDI before insulin pump, years	4 (2-8)	2 (2-7)	0.60
Hypothyroidism, n (%)	6 (15.8)	8 (19.5)	0.89

CSII, continuous subcutaneous insulin infusion; $HbA_{1c,}$ hemoglobin A_{1c}; BMI, body mass index; MDI, multiply insulin injection
*For the comparison of patients with LH number < 5 and ≥ 5.

Supplemental Table 3. The comparison of the number of any lipohypertrophic (LH) lesions according to the diagnostic method in type 1 diabetes (T1DM) patients (n=79).

Region	Visual assessment	Palpation	Physical examination (visual assessment and palpation)	Ultrasonography	*P**
All, n	72	134	152	372	<0.0001
Abdomen, n	62	92	107	189	0.004
Arms, n	4	8	8	31	0.06
Buttocks, n	3	19	19	69	0.01
Tights, n	3	19	21	78	0.17
Subscapula, n	-	-	-	5	0.32

*For the comparison of physical examination and ultrasonography.

Supplemental Table 4. The comparison of the number of LH lesions and LH severity score according to the type of infusion set used by T1DM patients.

	**Patients using Teflon cannulas, n=71***	**Patients using steel cannulas, n=7**	***P*****
LH number, mean ± SD	4.8 ± 3.4	4.9 ± 1.8	0.64
LH severity score, mean ± SD	5.0 ± 2.0	5.3 ± 1.0	0.78

* Including three patients who used Teflon and steel canula interchangeably. The information about one patient is missing.

** For the comparison of patients using Teflon versus steel cannulas.

Supplemental Table 5. The comparison of the number of LH lesions and LH severity score according to the insulin used by T1DM patients.

	**Patients treated with insulin lispro, n=52**	**Patients treated with insulin aspart, n=21**	**Patients treated with insulin glulisine, n=6**	***P****
LH number, mean ± SD	4.0 ± 2.7	4.7 ± 3.6	5.0 ± 3.5	0.47
LH severity score, mean ± SD	4.5 ± 1.9	5.0 ± 2.5	5.1 ± 1.9	0.47

*For the comparison of patients using insulin lispro versus insulin aspart versus insulin glulisine.
